# Value-Added Products Derived from Poly(ethylene terephthalate) Glycolysis

**DOI:** 10.3390/molecules29174261

**Published:** 2024-09-08

**Authors:** Simona Zahova, Pencho Tuleshkov, Kolio Troev, Violeta Mitova

**Affiliations:** Institute of Polymers, Bulgarian Academy of Sciences, Sofia 1113, Bulgaria; s.zahova@polymer.bas.bg (S.Z.); pen.tul@polymer.bas.bg (P.T.); ktroev@polymer.bas.bg (K.T.)

**Keywords:** PET glycolysis, glycolysis products, phosphorylation, polycondensation process, value-added products

## Abstract

Among polymer wastes, poly(ethylene terephthalate) (PET) is the most important commercial thermoplastic polyester. Less than 30% of total PET production is recycled into new products. Therefore, large amounts of waste PET need to be recycled. We describe a feasible approach for the direct application of the glycolysis products of PET (GP-PET), without further purification, for the synthesis of value-added products. It was established that GP-PET is valorized via phosphorylation with phenylphosphonic dichloride (PPD), as well as with trimethyl phosphate (TMP). When PPD is used, a condensation reaction takes place with the evolution of hydrogen chloride. During the interaction between GP-PET and TMP, the following reactions take place simultaneously: a transesterification with the participation of the hydroxyl group of GP-PET and the methoxy group of TMP and an exchange reaction between the ester group of GP-PET and the methyl ester group of TMP. The occurrence of the exchange reaction was confirmed by ^1^H, ^31^P, ^13^C NMR, and GPC analysis. Thermogravimetric analysis (TGA) revealed that the percentage of a carbon residual (CR) implies the possibility of using the end products as flame retardant (FR) additives, especially for polyurethanes as well as thermal stabilizers of polymer materials or Li-ion cells.

## 1. Introduction

In today’s modern society, plastic is an integral part of our daily lives. It has been shown that the most commonly used industrial polymers are not obtained from sustainable sources such as recycling, reuse processes, or renewable sources, and they are not bio-based or biodegradable [1]. Therefore, problems related to environmental pollution are inevitable. According to statistics, by 2022, about 10.5 Gt of plastics was produced worldwide, of which 6.5 Gt were scattered as waste [2]. Among these wastes, poly(ethylene terephthalate) (PET) is the most significant commercial thermoplastic polyester. Thanks to its excellent properties, such as thermal stability, mechanical strength, low gas permeability, and nontoxic nature, PET is a widely used polymer in the textile industry and in the production of food and beverage packaging. This polyester has been used in many electronic and electrical applications, especially those requiring high-temperature performance. By 2025, the global demand for the material is expected to reach 22.36 million tons [3]. On the other hand, 95% of the produced PET is disposed of as waste within a year, which is due to its short lifespan, as it is mainly used for packaging, and in most countries, <30% of discarded PET is recycled [4]. To solve this problem, researchers and chemical engineers have focused their efforts on recycling PET waste into high-value-added products. Among the possible recycling techniques, the most acceptable method that follows the principle of sustainable development is chemical recycling, mainly because it can lead to the formation of monomers/oligomers from which the polymer was made [5]. Bis(2-hydroxyethyl) terephthalate (BHET) obtained from the glycolysis process may be utilized as a starting material for a new synthesis of PET, rigid polyurethane [6], and bio-resorbable polyester [7], while the oligomers can be used to make polymers [8,9], hydrophobic dyestuffs [10], textile auxiliaries [11], water-soluble polyester coatings [12], and polymeric plasticizer [13].

Polymer materials are characterized by increased flammability. This can seriously endanger human life, cause much property damage, and limit the application of polymers in many fields. This problem has drawn the attention of researchers, producers, and government regulatory bodies to the creation of polymers with flame resistance [14]. Major developments for imparting flame resistance to polymers have been reviewed in the literature [15,16]. These methods include the physical addition of halogen/phosphorus-containing additives to the polymer or the chemical incorporation of FR monomers into the polymer chain. Typical FR additives can be classified into two categories—inorganic (metal hydroxides) and halogen-containing compounds [17,18]. Currently, they should meet requirements such as being non-toxic and environmentally friendly [19]. During combustion, halogen-containing additives emit toxic gases, making them not environmentally friendly, which is why phosphorus-based additives are broadly applicable [20]. From this class of FRs, polyphosphonates [21] and TMP [22] are often used.

PPD is used as a starting monomer in the synthesis of poly(arylphosphonates). These polymers have good FR, as indicated by their high values of limited oxygen index (LOI) of 50–60 [23]. Polyphosphonates synthesized by the polycondensation of PPD with poly (ethylene glycol) 12,000 with and without bisphenol A show good LOI values (in the range 28–38) [24].

TMP is used in a variety of industrial processes, including as an FR additive, solvent, and methylating agent for chemical reactions, as a fiber color inhibitor, as an intermediate for pesticides, and as a polymerization catalyst in industry and pharmaceuticals [25]. TMP is also used as a thermal stabilizer for the production of PET [26]. It has been shown that the thermal stability of Li-ion cells can be improved by using TMP-containing electrolytes [27].

The aim of the present research is to find new fields of application for the products of the glycolysis of PET, namely, additives for polymers, giving them new properties or improving their existing ones. In the present experiment, in order to develop a circular production model, reduce the cost of product separation and the drawbacks of plastic waste treatment, and increase the value of recycled products, we describe the results of a feasible approach for the direct application of the product of glycolysis of PET (GP-PET). GP-PET [28] is a well-defined mixture of monomers, dimers, trimers (and other oligomers), and ethylene glycol (EG) and can be used without further purification for the synthesis of phosphorus-containing compounds by polycondensation with PPD and TMP. The resulting products are phosphorus-containing oligomers. The use of oligomeric analogs of a polymer as an additive could be a good option because it would impart good compatibility between the oligomeric additive and the polymer. To the best of our knowledge, there are no previous works in the literature describing the valorization of the products of the glycolysis of PET via phosphorylation with PPD and TMP.

## 2. Results and Discussion

### 2.1. Interaction between GP-PET and PPD

**Scheme 1:**
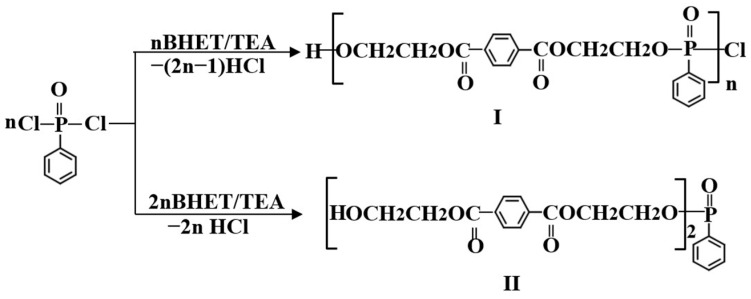
Interaction between BHET and PPD at molar ratios of 1:1 and 2:1.

The literature does not contain ^31^P NMR data for phosphorus atoms with surroundings, as in the expected reaction products obtained from the interaction between PPD and the products of PET glycolysis (GP-PET). An analysis of GP-PET [28] revealed that approximately 50% of its composition is BHET. Based on this, and for the purpose of the signal assignment, two model reactions between BHET (commercial product) and PPD were performed at molar ratio BHET/PPD = 1:1 and = 2:1 (Scheme 1) (details about the experimental procedure of the model reactions are reported in the Supplementary Materials).

#### 2.1.1. The Model Reactions—Interaction between BHET and PPD

**Figure 1:**
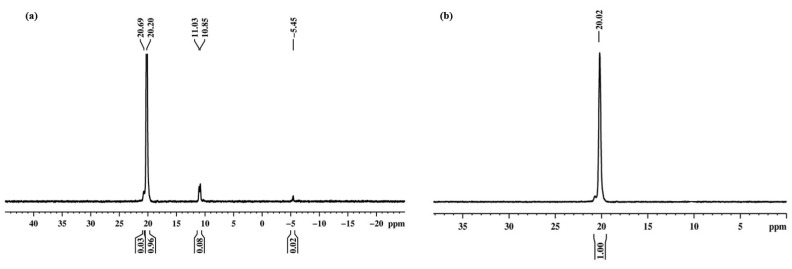
The ^31^P{H} NMR spectra of the reaction product at molar ratios of (**a**) 1:1 (BPClTEA) and (**b**) 2:1 (2BPClTEA).

In the ^31^P{H} NMR spectrum of the reaction product (BPClTEA) obtained at a molar ratio of 1:1 (Figure 1a), there are signals at 20.69 ppm, 20.20 ppm, 11.03 ppm, 10.85 ppm, and −5.45 ppm with integral intensities 0.03, 1.00, 0.08, and 0.02, respectively. The signals at 20.69 ppm and 20.20 ppm are characteristic of the mono and diesters of phenylphosphonic acid [29,30]. The signals at 11.03 ppm and −5.45 ppm should be attributed to phenylphosphonic acid [31] and pyrophosphonate structures [31], respectively. The ^31^P{H} NMR spectrum of the reaction product (2BPClTEA) obtained at a molar ratio of 2:1 (Figure 1b) showed a signal at 20.02 ppm. The signals at 20.20 ppm (molar ratio 1:1) and 20.02 ppm (molar ratio of 2:1) have a significantly stronger integral intensity, which gives us reason to attribute them to the phosphorus atom in the repeating unit of **I** and in di[bis(2-hydroxyethylterephthalate)] phenylphosphonate **II**. The phosphorus atoms in both products have the same surroundings.

In the ^1^H NMR spectrum of BPClTEA (Figure S1), the signal at 3.89 ppm, a triplet with a coupling constant ^3^J(H, H) = 4 Hz, refers to HOC*H*_2_CH_2_-; the triplet at 4.44 ppm relates to methylene protons of HOCH_2_C*H*_2_-OC(O). In the range of 7.29–8.02 ppm, aromatic protons should be attributed to the hydrogen atoms of BHET and the aromatic nucleus of PPD. Signals in the region 4.34–4.24 ppm, representing multiplets, should refer to -CH_2_CH_2_O-P(O)-OCH_2_CH_2_- protons. The signal at 3.03 ppm for the proton of the end hydroxyl group *H*O-CH_2_CH_2_- was also observed. The same characteristic signals occur in the ^1^H NMR spectrum of product 2BPClTEA (Figure S2). The ^13^C NMR data of BPClTEA (Figure S3) indicate characteristic signals at 59.86 ppm (HO*C*H_2_CH_2_-), 66.95 ppm (HOCH_2_*C*H_2_-), and aromatic carbon atoms of BHET and PPD in the range of 125.01–132.52 ppm. Resonances at 164.84 ppm and 164.25 ppm for the carbonyl group were also observed. New signals appeared at 62.98 ppm, a doublet with a coupling constant ^3^J(P, C) = 6 Hz, typical for -CH_2_*C*H_2_O-P(O)- carbon atom, and at 66.05 ppm for -*C*H_2_CH_2_O-P(O)-. Based on the NMR (^1^H, ^13^C, ^31^P{H}) data, we assume that the product of the interaction between BHET and PPD at a molar ratio 1:1 has a structure that coincides with the one presented in Scheme 1 (Scheme 1, product I).

The above results give us reason to assume that if the signal at 20.20 ppm (integral intensity 0.96) in the ^31^P NMR spectrum is for the phosphorus atom in the repeating units, and that at 20.69 ppm (integral intensity 0.03) is for the phosphorus atom in the end unit, then the molecular mass (M_n_) of polyphenylphosphonate is 12,032 g/mol (n = 32, molecular weight of the repeating unit 376).

#### 2.1.2. Interaction between GP-PET and PPD at a Molar Ratio of 1:1

**Scheme 2:**
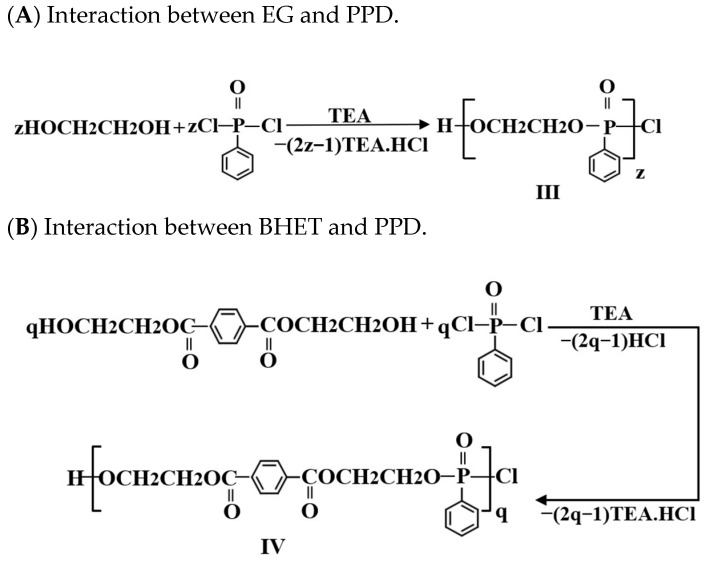
Interaction between (**A**) EG and PPD and (**B**) BHET and PPD.

A total of 10.000 g of PG-PET, including 48.78% BHET; dimer, 20.89%; trimer, 10.96%; and EG, 19.37%, reacts with 11.075 g (0.0568 mol) of PPD. EG, 0.0312 mol, and BHET, 0.0192 mol, have the highest molar concentrations, while the molar concentrations of the dimer and trimer are less by one order of magnitude. This gives us reason to assume that the following two main reactions occur simultaneously (Scheme 2).

The interaction between **III** and **IV** leads to the formation of the copolymer **VI**.

The total molar concentration of EG (0.0312 mol) and BHET (0.0192 mol) is 88.7% from the molar concentration of GP-PET (0.0568 mol). Products **III** and **IV**, which are based on EG and BHET, are approximately 90% of the weight of the reaction product, i.e., these are the primary products of interaction. The ^1^H NMR spectrum of the reaction product (Figure S4) showed a signal at 10.97 ppm, which is characteristic of P-OH protons. The signal at 8.02 ppm is characteristic of the aromatic protons of PET. The aromatic protons of PPD are in the range of 7.33–7.94 ppm. The additional signals at 4.63 ppm are attributed to the methylene protons in the segments -C(O)O-CH_2_-CH_2_-OCO-, which are due to the presence of dimers and trimers in GP-PET. The signals at 3.88 ppm and 4.43 ppm are assigned to the methylene protons adjacent to the hydroxyl group in the BHET unit (-CH_2_-OH) and C(O)O-CH_2_. The multiplets at 4.38–4.19 ppm refer to the methylene protons of P(O)O-C*H*_2_CH_2_ and P(O)O-CH_2_C*H*_2_. At 3.00 ppm, there is a signal for the proton of the *H*OCH_2_-CH_2_ structure.

**Figure 2:**
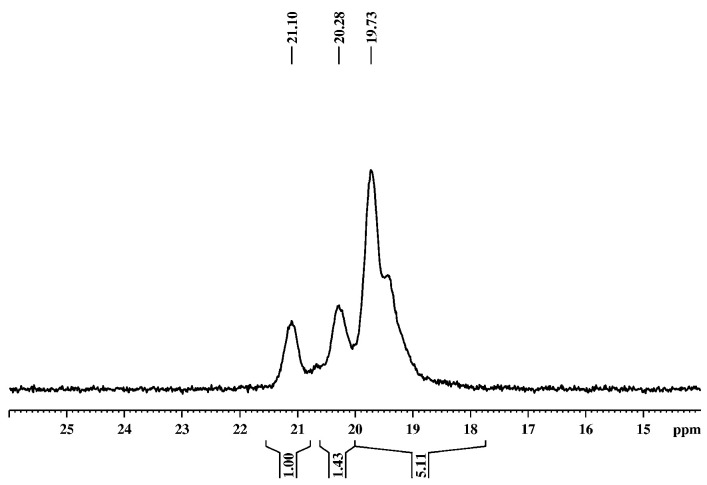
The ^31^P{H} NMR spectrum of GP-PET/PPD.

In the ^31^P{H}NMR spectrum (Figure 2) of the reaction product, there are signals at 19.73, 20.28, and 21.10 ppm with integral intensities of 5.11, 1.43, and 1.00, respectively. Three types of phosphorus atoms must exist, namely, the phosphorus atom in the repeating unit of product **III**, the repeating unit of product **IV**, and the terminal unit. Based on the literature data [29,30], the signals at 19.73 ppm and 20.28 ppm can be assigned to the phosphorus atom in the repeating units, while the one at 21.10 ppm can be assigned to a phosphorus atom in the terminal unit. The quantitative composition of the degradation product suggests that the signal at 19.73 ppm can be attributed to the phosphorus atom in the repeating units of product **III** and that at 20.28 ppm can be attributed to the phosphorus atom in the repeating unit of product **IV**. The number average molecular mass of the phosphorylated product, calculated based on the data from the ^31^P{H}NMR spectrum, is 1447 g/mol.

**Scheme 3:**
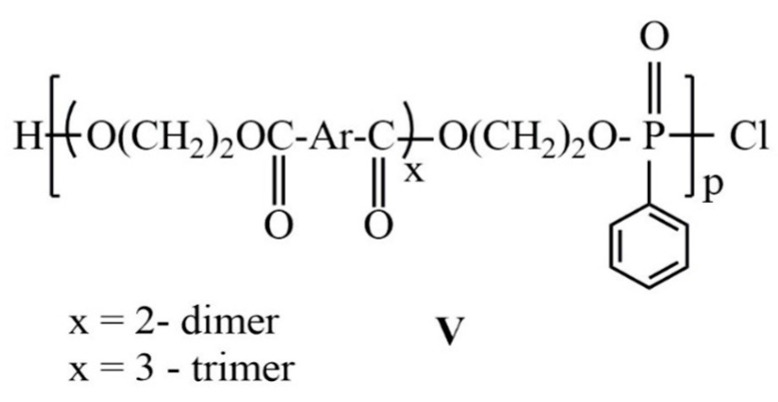
Reaction products of the interaction between the dimer and trimer and PPD.

**Scheme 4:**
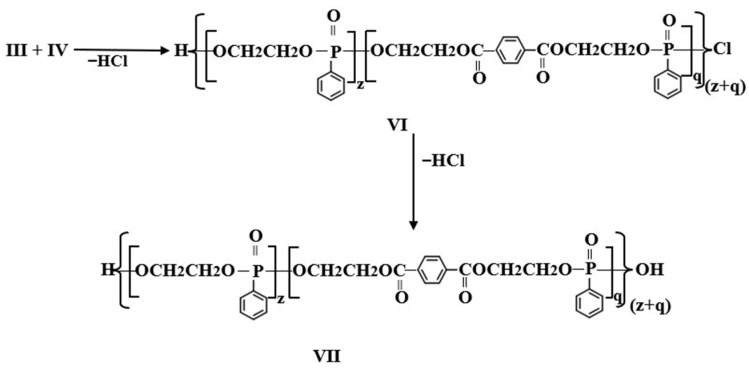
Interaction between **III** and **IV**.

In the ^13^C NMR spectrum of the reaction product (Figure S5), there are signals at 67.97 ppm for HO-CH_2_*C*H_2_O- and 60.90 ppm for HO-*C*H_2_CH_2_O-. There are signals at 63.00 ppm for -C(O)O-CH_2_-*C*H_2_-OCO-, which are due to the presence of dimers and trimers in the product; at 133.99–128.40 ppm for the aromatic carbon atoms of BHET and PPD residues; and at 165.55 ppm and 166.03 ppm for C=O carbon atoms. New signals appear at 66.90 ppm for -P(O)O-CH_2_*C*H_2_-O(O)C- and a doublet at 63.57 ppm with ^2^J(P,C) = 5.7 Hz, characteristic of the -CH_2_*C*H_2_O(O)P- carbon atom. The NMR data for reaction products of the dimer and trimer with PPD, **V** (Scheme 3), will be the same as those for products **III** and **IV** (Scheme 4) because the substituents attached to the phosphorus atom are the same. The data from NMR spectroscopy confirm the proposed structures.

### 2.2. Interaction between GP-PET and TMP

#### 2.2.1. Transesterification of TMP with Commercial BHET

The model reaction of BHET and TMP at a molar ratio of 1:2 was carried out at 190 °C for 5 and 9 h (details about the experimental procedure of the model reaction are reported in the Supplementary Materials).

In the ^31^P{H} NMR spectrum of the reaction product obtained after 5 h of heating, BTMP5 (Figure S6), there are signals (δ, ppm/integral intensity) at 2.63 (1.00), 2.30 (2.48), 1.48 (0.48), 1.17 (0.75), and 0.05 (0.30), which are characteristic of phosphate structures. The main signals are at 2.63 ppm and 2.30 ppm in a ratio of 1:2.48 (28.70%, 71.30%). In the ^31^P NMR spectrum of the product (Figure S7), the signals represent multiplets of nine lines (^3^J (P, H) = 11.38 Hz), which gives information about the phosphorus surrounding atoms.

In the ^31^P{H} NMR spectrum of the reaction product obtained after 9 h of heating, BTMP9 (Figure S8), the signals are at 2.70 ppm and 2.29 ppm in a ratio of 1.00:2.32 (30.10%, 69.90%). From the ^31^P NMR analysis of the same product (Figure S9), it is clear that the signals are multiplets of nine lines with a coupling constant ^3^J (P, H) = 11.74 Hz.

The data from the ^31^P{H} NMR analysis show that the additional increase in the reaction time does not lead to significant changes in the content of the reaction products. The intensity of the signal at 2.70 ppm increases from 28.70% up to 30.10%. The presence of two signals gives us reason to assume that in the reaction mixture, there are two phosphorus-containing compounds with different amounts but with a very similar structure of the substituents at the phosphorus atom. 

In the ^1^H NMR spectrum of BTMP5 (Figure S10), the signal at 3.34 ppm should be attributed to the proton of the OH group (*H*OCH_2_-CH_2_- structure). The doublets at 3.66 ppm and 3.69 ppm with ^3^J(P,H) = 12 Hz are characteristic of POC*H*_3_ protons and display integral intensities of 1.42 and 3.50, respectively, in a ratio of 1.00:2.47, which is the same as the ratio of the integral intensities of the signals for the phosphorus atoms, i.e., 1.00:2.48.

The signals in the region 4.29–4.42 ppm should be attributed to -C(O)OCH_2_- and P(O)OCH_2_- protons. The signals at 8.01 ppm and 8.03 ppm are assigned to the aromatic protons. In the ^1^H NMR spectrum, there is a new signal at 3.86 ppm, a singlet, which is characteristic of the methyl protons of the ester group CH_3_OC(O)-Ar-. The reason for assigning this signal to these protons is the fact that in the starting compounds, BHET and TMP, there are no protons whose signals are singlets in this region. Additionally, the signal for the methyl protons of dimethyl terephthalate is at 3.94 ppm [32]. The ^1^H NMR spectrum of BTMP9 (Figure S11) contains the same signals as the 5 h heating product. The ratio of the integral intensities of the signals for POCH_3_ protons is almost the same, from 1:2.48 to 1:2.45.

In the ^13^C NMR spectrum of BTMP9 (Figure S12), there are signals at 52.42 ppm, a singlet; 54.12 ppm, d,^2^J(P,C) = 6.0 Hz, and 54.46 ppm, d, ^3^J(P,C) = 6.0 Hz, which should be attributed to P-O*C*H_3_ carbon atoms; 59.04 ppm for HO*C*H_2_ carbon atoms; 63.81 ppm, d, ^2^J(P,C) = 5.7 Hz, which should be assigned to P(O)O*C*H_2_ carbon atoms, and 70.41 ppm for *C*(O)OCH_2_- carbon atoms; and at 133.70 ppm and 129.65 ppm for aromatic carbon atoms. There are also two signals for the carbonyl carbon atom at 165.43 and 166.16 ppm (*C*=O carbon atoms). The ^13^C NMR spectrum shows the presence of two types of P-OCH_3_ carbon atoms, which is in agreement with the ^1^H NMR and ^31^P NMR spectroscopy results. The singlet at 52.42 ppm can be attributed to the carbon atom of the methyl ester group (*C*H_3_OC(O)-) since the signal for this carbon atom of dimethyl terephthalate is at 52.39 ppm [32].

**Scheme 5:**
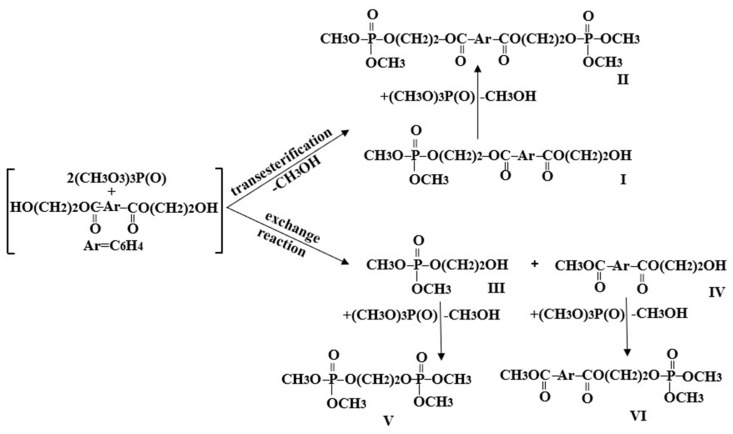
Interaction between BHET and TMP.

A phosphorus atom whose signal in the ^31^P NMR spectrum is a multiplet of nine lines can be obtained as a result of a transesterification reaction of TMP and BHET, and it is also as a result of an exchange reaction between the ester group of BHET and the methoxy group of TMP. The signals for the phosphorus atoms of the products of transesterification (compounds **I** and **II**, Scheme 5) should be at the same shift in the spectrum because their surroundings are the same. The signal for the phosphorus atom of dimethyl(2-hydroxyethyl) phosphate **III** (Scheme 5) should not coincide with that of compounds **I** and **II**, since there is a difference in the substituents—in dimethyl(2-hydroxyethyl) phosphate **III**, the substituent is OCH_2_CH_2_OH, while in **I** and **II**, it is OCH_2_CH_2_OC(O)-Ar-C(O)OCH_2_CH_2_OH. A methyl ester group -CH_3_OC(O)- is formed as a result of the exchange reaction. It is known that the alkoxy groups of H-phosphonic and the phosphoric acids participate in exchange reactions with amide [33], urethane [34], and carbonate [35] groups. We assume that the reaction between BHET and TMP proceeds according to the following reaction scheme (Scheme 5).

Under these reaction conditions, two reactions take place simultaneously as follows: transesterification between TMP and BHET and an exchange reaction between TMP and BHET. In the first stage of transesterification, product **I** is formed, which, in the second stage, is converted into product **II**. Since the exchange reaction proceeds at a lower rate compared with the transesterification reaction [33,34,35], it can be assumed that the signal at 2.29 ppm (Figure S8) should be related to the phosphorus atom in product **II** and that at 2.70 ppm should be related to the phosphorus atom in dimethyl(2-hydroxyethyl) phosphate **III**. Its content based on ^31^P{H} NMR is 30.10%. The content of methyl (2-hydroxyethyl) terephthalate **IV** is the same. Transesterification of TMP with dimethyl(2-hydroxyethyl) phosphate **III** and methyl (2-hydroxyethyl) terephthalate **IV** leads to the formation of compounds **V** and **VI** (Scheme 5). The NMR data suggest that the main products of the reaction of BHET (commercial product) with TMP are **II**, with a content of 70%, and **V** and **VI** with a content of 30%.

#### 2.2.2. Interaction between GP-PET and TMP at a Molar Ratio of 1:2

**Figure 3:**
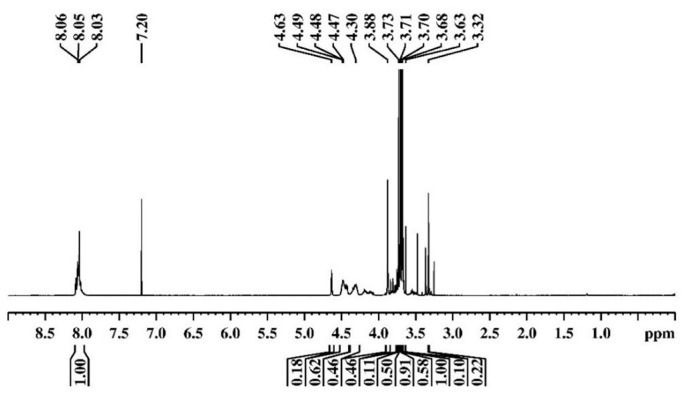
The ^1^H NMR spectrum of GP-PET/TMP.

**Figure 4:**
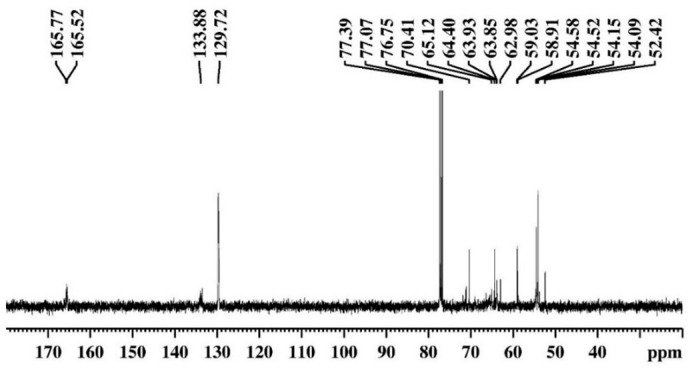
The ^13^C NMR spectrum of GP-PET/TMP.

It was found that the content of GP-PET is BHET, 48.78%; dimer, 20.89%; trimer, 10.96%; and EG, 19.37% [28]. A reaction between GP-PET and TMP was carried out at a temperature of 190 °C for 3 h at a molar ratio of 1:2. The reaction product was characterized by ^1^H, ^31^P, and ^13^C NMR techniques. The data from the ^1^H and ^13^C NMR analysis (Figure 3 and Figure 4) are similar to those of the reaction product from the interaction between commercial BHET and TMP. The presence of signals at 3.88 ppm in the ^1^H NMR spectrum and 52.42 ppm in the ^13^C NMR spectrum confirms the assumption that an exchange reaction also takes place in the reaction mixture of GP-PET /TMP.

**Figure 5:**
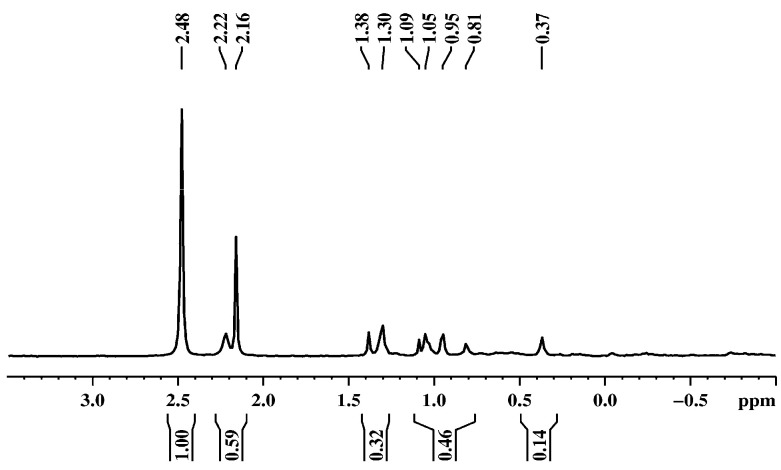
The ^31^P{H} NMR spectrum of GP-PET/TMP.

**Figure 6:**
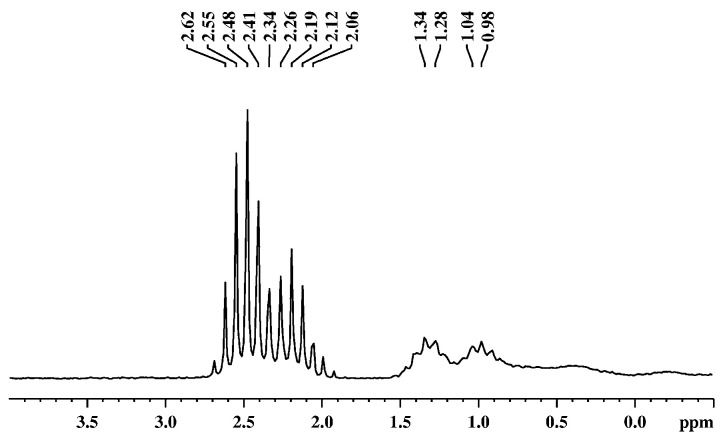
The ^31^P NMR spectrum of GP-PET/TMP.

In the ^31^P{H} NMR spectrum of GP-PET/TMP (Figure 5), the main signals (δ, ppm/integral intensity) are at 2.48 (1.00) and 2.16 (0.59). In the ^31^P NMR spectrum of GP-PET/TMP (Figure 6), the signals represent multiplets of nine lines with a coupling constant ^3^J(P,H) = 11.34 Hz.

**Scheme 6:**
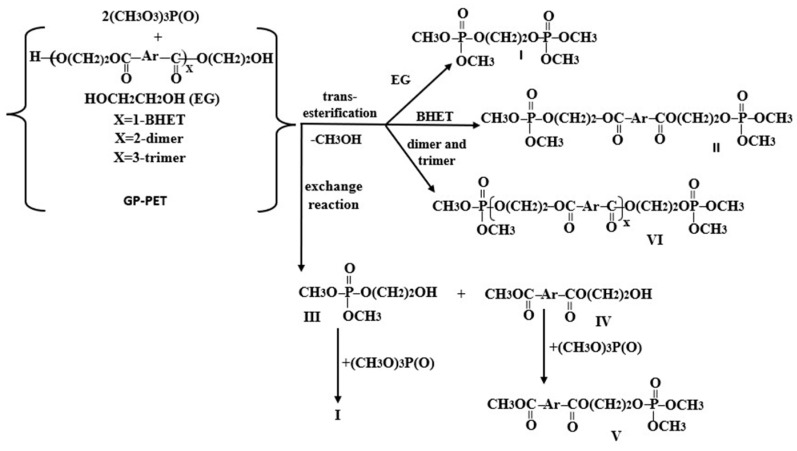
Interaction between GP-PET and TMP.

The content of 10.000 g GP-PET is BHET (0.0192 mol), dimer (0.0047 mol), trimer (0.0017 mol), and EG (0.0312 mol). The molar concentration of EG is the highest—1.6 times higher than that of BHET, 6.6 times higher than that of the dimer, and 18 times higher than that of the trimer. The GPC analysis (Figure S13) shows that the reaction mixture contains products with a molecular weight (Mw) of 221, 297, 433, 628, and 835. Based on the ratio of the molar concentrations of the components of the glycolysis product and TMP, and the data from the GPC analysis, we propose the following reaction scheme for the interaction between GP-PET and TMP (Scheme 6).

According to the proposed reaction scheme, two reactions occur simultaneously in this interaction as follows: a transesterification reaction, whose products are **I**, **II**, **V**, and **VI**, and an exchange reaction, whose products are **III** and **IV**.

The GPC analysis indicates that GP-PET contained products with molecular weights (Mw) of 221, 297, 433, 628, and 835. The molecular mass of 221 should be attributed to product **IV** (Mw = 224), which confirms the progress of the exchange reaction. The molecular weight of 297 should be assigned to product **I** (Mw = 278), and the molecular weight of 433 should be assigned to product **II** (Mw = 470). The molecular weights of 628 and 835 should be attributed to products **VIa** (x = 2) (Mw = 662) and **VIb** (x = 3) (Mw = 856).

Table 1 summarizes the data for the molecular weights (Mn and Mw) and polydispersity index (PDI) of the products from the interaction between GP-PET and TMP.

### 2.3. Evaluation of the Thermal Characteristics

Molecules containing phosphorus atoms in their architectures (inorganic phosphate and organophosphorus compounds) are used as flame retardants. These substances support inhibitory layer formation on the surface of a polymeric matrix during combustion and reduce the contact area between the polymer and oxidizing agents. During the combustion reaction, a carbon structure is formed, which is a solid layer. Decomposition of the phosphorus-containing mixtures generates radicals of PO·, P_2_, and P that have the ability to capture the radicals of H·, O·, and HO·. These facts result in the formation of high quantities of CR after thermal degradation at high temperatures even in an inert atmosphere [36]. It is well known that CR is a very important characteristic for determining their abilities as flame retardant additives since CR can promote an intumescent effect in the polymeric matrix and form a physical limitation to oxygen therein. In summary, the CR mass produced after thermal decomposition of phenylphosphonate compounds is directly related to the amounts of phosphorus elements in the polymer chain [37].

From the TGA curve of product BPClTEA (Figure S14), it can be seen that the decomposition of the sample proceeds in the following three steps: during the first stage of degradation from about 90 to 200 °C, the weight lost is about 9%; in the second stage from 200 to 390 °C, the decomposition rate is higher—31%; and in the third stage from 390 to 500 °C, the weight lost is about 43%. At 800 °C, the carbonized residue is about 17%. From the TGA analyses of BTMP9 (Figure S15), it is evident that the degradation of the material takes place in three stages as follows: at 350 °C, which refers to a weight loss of around 57%; in the second (at 450 °C) and third (at 700 °C) stages, the losses of the material are around 28%. The CR at 800 °C is nearly 15%.

**Figure 7:**
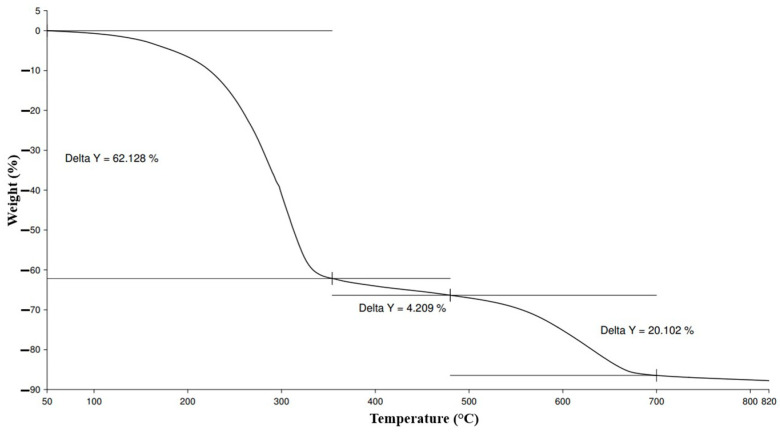
TGA curve of GP-PET/TMP.

From the TGA data of the reaction product from the interaction between GP-PET and TMP (Figure 7), it is obvious that the decomposition of the sample proceeds in three stages as follows: the first stage is at 360 °C, which refers to the weight loss of 62%; in the second (at 500 °C) and third (at 700 °C) stages, the losses are around 25%. The remaining residue at 800 °C is about 13%.

**Figure 8:**
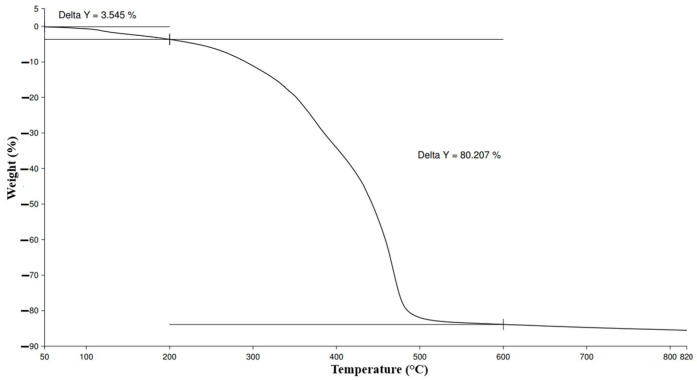
TGA curve of GP−PET/PPD.

From the TGA curve of the reaction product of the interaction between GP-PET and phenylphosphonic dichloride (GP-PET/PPD) (Figure 8), it is established that the thermal degradation of the product takes place in three stages. In the first stage, insignificant losses (~3.5%) are observed at a temperature of about 200 °C. In the next two phases from 250 to 400 °C and from 400 to 600 °C, the losses of material (nearly 80%) indicate the thermal decomposition of the phosphor-containing product. The thermogram also shows that after heating to 800 °C, the amount of CR is approximately 17%.

The quantity of the remaining residue of the phosphorylated products is similar to others flame retardants applied as additives to polymers. The CR values of the products synthesized in the present study are comparable to data reported for other phosphorylated materials [38,39] and show the possibility for their potential use as flame retardants.

## 3. Materials and Methods

### 3.1. Materials

The product of glycolysis of PET (GP-PET) obtained and reported in our earlier experiment [28] had the following composition:

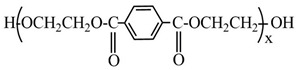

BHET (x = 1), 48.78%, Mw = 254.24 g/mol; dimer (x = 2), 20.89%, Mw = 446.4 g/mol; trimer (x = 3), 10.96%, Mw = 638.56 g/mol; and EG, 19.37%, Mw = 62.07 g/mol. Phenylphosphonic dichloride (PPD) was purchased from Merck, Germany (Purity ≥ 97%); trimethyl phosphate (TMP) was supplied by Janssen Chimica (Purity ≥ 99%); and bis(2-hydroxyethyl) terephthalate (BHET) and dry tetrahydrofuran (THF) were purchased from Sigma Aldrich, USA. All these materials were used without further purification. Triethylamine (TEA) (Sigma Aldrich) was distilled prior to use.

### 3.2. Measurements

Gel permeation chromatography (GPC) analysis was prepared on a Shimadzu Nexera, equipped with a differential refractive detector RID-20A and the following column set: PSS SDV Linear M (300 mm × 8 mm × 5 μm), PSS SDV 100 Å (300 mm × 8 mm × 5 μm), PSS SDV 50 Å (300 mm × 8 mm × 5 μm), at the following measurement conditions: column temperature of 45 °C, THF as a solvent, mobile phase flow of 1 mL/min injection, temperature of 25 °C, and injection volume of 100 μm.

The ^1^H, ^31^P{H}, ^31^P, and ^13^C NMR spectra were obtained with a Buker Avance Neo 400 spectrometer in CDCl_3_. TGA analyses were carried out on a LINSEIS thermal analyzer, model STA PT1600, in argon medium at the heating of 40–800 °C and a flow rate of 50 mL/min.

### 3.3. Synthesis of Polyphosphonate GP-PET/PPD

First, 10.000 g (total number of moles of hydroxyl-containing compounds 0.0568 mol) of GP-PET and 760 mL THF were placed into a 1 L tree-necked round bottom flask equipped with a magnetic stirring bar, a capillary for argon purging, a thermometer, a dropping funnel, and a reflux condenser at room temperature with vigorous stirring for 12 h until GP-PET was completely dissolved. Then, TEA (11.495 g, 0.1136 mol) was added to the mixture. After that, a solution of PPD (11.075 g, 0.0568 mol) in 15 mL THF was added dropwise into the flask for 40 min under constant stirring and cooling. After dripping, the mixture reacted overnight at ambient temperature. Then, the reaction was carried out at 50 °C for 8 h, and the mixture was cooled down to room temperature. The precipitate formed, i.e., triethylamine hydrochloride (TEA.HCl), was separated by filtration. The product was isolated via evaporation of the solvent on a vacuum rotary evaporator and dried under reduced pressure. It was washed with distilled water several times and dried at 50 °C under reduced pressure for 24 h to obtain a soft, wax-like product (18.040 g, yield: 80.2%). It was labeled **GP-PET/PPD** and characterized by ^1^H, ^31^P{H}, ^13^C NMR, and TG analyses. 

The ^1^H NMR (400 MHz, CDCl3) δ (ppm) was as follows: 10.97, s, P-O*H*; 8.02–7.33, m, Ar-*H*; 4.63, s, -C(O)O-C*H*_2_-C*H*_2_-OCO-, dimers and trimers; 4.43, s, C(O)O-C*H*_2_; 4.38–4.19, m, P(O)O-C*H*_2_C*H*_2_; 3.88, s, -C*H*_2_-OH; 3.00, s, *H*-OCH_2_-CH_2_; ^31^P{H} NMR (400 MHz, CDCl_3_) δ (ppm): 21.10, 20.28, 19.73; ^13^C NMR (400 MHz, CDCl_3_) δ (ppm): 166.03 and 165.55, *C*=O; 133.99–128.40, aromatic carbon atoms of BHET and PPD; 67.97, OH-CH_2_*C*H_2_O-; 66.90, -P(O)O-CH_2_*C*H_2_-O(O)C-; 63.57 d, ^3^J(P,C) = 5.7 Hz, CH_2_*C*H_2_O(O)P-; 63.00, -C(O)O-*C*H_2_-*C*H_2_-OCO- dimers and trimers, 60.90 OH- *C*H_2_CH_2_O-.

### 3.4. Synthesis of Polyphosphate GP-PET/TMP

First, 10.000 g (total number of moles of hydroxyl-containing compounds 0.0568 mol) of GP-PET and 15.913 g (0.1136 mol) of TMP were mixed in a three-necked round bottom flask equipped with an argon purging capillary, a magnetic stirrer, a condenser, and thermometer. The reaction was carried out at 190 °C with permanent stirring for 3 h. The reaction completion indicator was the termination of the release of methanol. The content of the flask was allowed to cool down to ambient temperature. The product was dried under reduced pressure to constant weight. A soft, brownish wax-like product was obtained. It was labeled **GP-PET/TMP** and characterized by ^1^H, ^31^P{H}, ^31^P, ^13^C NMR, GPC, and TG analyses. The reaction completion rate was 92.50% (24.012 g).

The ^1^H NMR (400 MHz, CDCl_3_) δ (ppm) was as follows: 10.85, s, P-O*H*; 8.06–8.03, m, Ar-*H*; 4.63, s, -C(O)O- C*H*_2_-C*H*_2_-OCO-; 4.49–4.30, m, -C(O)OC*H*_2_- and P(O)O-C*H*_2_-CH_2_-; 3.88, s, C*H_3_*OC(O)-Ar-; 3.72, d, ^3^J(P,H) = 12 Hz, P(O)O-C*H*_3_; 3.70, d, ^3^J(P,H) = 12 Hz, P(O)O-C*H*_3_; 3.32, s, -CH_2_CH_2_-O*H*; ^31^P{H} NMR (400 MHz, CDCl_3_) δ (ppm): 2.48, 2.16; ^31^P NMR (400 MHz, CDCl_3_) δ (ppm): 2.48, m, ^3^J(P,H) = 11.34 Hz, -(CH_2_)_2_O(O)P(OCH_3_)_2;_ 2.16, m, ^3^J(P,H) = 11.34 Hz, HO(CH_2_)_2_O(O)P(OCH_3_)_2;_ ^13^C NMR (400 MHz, CDCl_3_) δ (ppm): 165.77 and 165.72, *C*=O; 133.88 and 129.72, aromatic carbon atoms; 70.41, C(O)O*C*H_2_-; 63.89, d, ^3^J(P,C) = 5.7 Hz, P(O)O-*C*H_2_CH_2_; 62.98, -C(O)O-*C*H_2_-*C*H_2_-OCO- dimers and trimers; 59.03, HO*C*H_2_; 54.55, d, ^3^J(P,C) = 6.0 Hz, P-O*C*H_3_; 54.12, d, ^3^J(P,C) = 6.0 Hz, P-O*C*H_3_; 52.42, *C*H_3_OC(O)-Ar-.

## 4. Conclusions

In this study, the possibility of valorizing GP-PET was successfully proven, yielding high-value products. It was found that GP-PET can be directly used as a source for phosphorus-containing oligomers and monomers via a polycondensation reaction with phenylphosphonic dichloride or transesterification and an exchange reaction with trimethyl phosphate. The polycondensation reaction between GP-PET and phenylphosphonic dichloride proceeds in the presence of triethylamine at a molar ratio of 1:1 to obtain a soft, wax-like oligomer after heating at 50 °C for 8 h. In the repeating unit, the oligomer contains a phosphorus atom and an aromatic group, which are expected to increase fire retardancy. The thermogravimetric analysis revealed that the char residue of GP-PET/PPD is around 17%, which suggests the possibility of its application as a flame retardant of polymers (polyurethanes, PET). The interaction between GP-PET and trimethyl phosphate at molar ratio of 1:2 at 190 °C resulted in the formation of triesters of phosphoric acid, possessing reactive groups, which allows new phosphorus-containing products to be obtained on their basis. In addition, triesters of phosphoric acid have been used as thermal stabilizers of polymer materials or Li-ion cells [40,41,42].

## Figures and Tables

**Table 1:** Molecular weights and PDI for the resulting products from the interaction between GP-PET and TMP.

**Product**	**Mn**	**Mw**	**PDI**
**I**	295	297	1.005
**II**	428	433	1.011
**IV**	219	221	1.008
**VIa**	623	628	1.008
**VIb**	831	835	1.005

Product numbers are described relative to Scheme 6.

## Data Availability

The data presented in this study are available on request from the corresponding author.

## Supplementary Materials

The following supporting information can be downloaded at https://www.mdpi.com/article/10.3390/molecules29174261/s1, Detailed experimental procedures: S1. Model reactions; S1.1. Interaction between BHET and PPD in the presence of the HCl acceptor at a molar ratio of 1:1 (model reaction 1). Product BPClTEA; S1.2. Interaction between BHET and PPD in the presence of the HCl acceptor at a molar ratio of 2:1 (model reaction 2). Product 2BPClTEA; S1.3. Interaction between BHET and TMP at a molar ratio of 1:2 (model reaction 3). Products BTMP5 and BTMP9. The ^1^H, ^31^P, ^13^C NMR spectra and GPC data of the products: Figure S1. The ^1^H NMR spectrum of BPClTEA; Figure S2. The ^1^H NMR spectrum of 2BPClTEA; Figure S3. The ^13^C NMR spectrum of BPClTEA; Figure S4. The ^1^H NMR spectrum of GP-PET/PPD; Figure S5. The ^13^C NMR spectrum of GP-PET/PPD; Figure S6. The ^31^P{H} NMR spectrum of BTMP5; Figure S7. The ^31^P NMR spectrum of BTMP5; Figure S8. The ^31^P{H} NMR spectrum of BTMP9; Figure S9. The ^31^P NMR spectrum of BTMP9; Figure S10. The ^1^H NMR spectrum of BTMP5; Figure S11. The ^1^H NMR spectrum of BTMP9; Figure S12. The ^13^C NMR spectrum of BTMP9; Figure S13. Molecular weight distribution according to GPC analysis of GP-PET/TMP; TGA curves of the model reactions: Figure S14. TGA curve of BPCTEA; Figure S15. TGA curve of BTMP9.
