# Supplementary material for: Value-Added Products Derived from Poly(ethylene terephthalate) Glycolysis
Source: Molecules. 2024 Sep 8;29(17):4261. doi: 10.3390/molecules29174261 (PMC11397719; doi:10.3390/molecules29174261)
Supplement: Supplementary file 1 [file molecules-29-04261-s001.zip › molecules-3178995-SI.pdf]

Supplementary materials

for

Value added products derived from poly(ethylene terephthalate) glycolysis

Simona Zahova, Pencho Tuleshkov, Kolio Troev and Violeta Mitova *

Institute of Polymers, Bulgarian Academy of Sciences, Sofia 1113, Bulgaria;

s.zahova@polymer.bas.bg (S.Z.); pen.tul@polymer.bas.bg (P.T.); ktroev@polymer.bas.bg (K.T.)

* Correspondence: mitova@polymer.bas.bg; Tel.: +359 2 9796637

Number of pages: 11

Number of figures:15

S1. Model reactions

S1.1. Interaction of BHET with PPD in the presence of HCl acceptor at a molar ratio 1:1 (model reaction 1). Product BPClTEA.

BHET (1.000 g, 0.0039 mol) and 75 ml THF were placed into a 100 ml tree-necked round bottom flask equipped with a capillary for inert gas purging, a magnetic stirring bar, a thermometer, a dropping funnel and a reflux condenser. The mixture was stirred at room temperature until BHET was completely dissolved. Then 0.789 g (0.0078 mol) TEA was added in to the mixture. After that a solution of PPD (0.760 g, 0.0039 mol) and 1 ml THF was added dropwise into the flask, which was continuously cooled during the process. After dripping finished, the mixed solution reacted overnight with constant stirring at ambient temperature. Then the reaction was carried out at 50°C for 8 h. The content of the flask was allowed to cool down to room temperature. The precipitate formed, triethylamine hydrochloride (TEA.HCl) was separated by filtration. The product was isolated via evaporation of the solvent on a vacuum rotary evaporator and dried under reduced pressure to get a semi-transparent, with a whitish color soft product (1.454 g, yield: 90.76%). It was labeled **BPClTEA** and characterized by ^{1}H, ^{31}P{H}, ^{13}C NMR and TG analyses.

^{1}H NMR (400 MHz, $CDCl_3$) δ (ppm): 8.02-7.29, m, Ar-*H*; 4.44, t, $HOCH_2CH_2$-OC(O), 4.34-4.24, m, –CH_2CH_2O-P(O)-OCH_2CH_2; 3.89, t, ^{3}J(H,H)=4 Hz, -HO$CH_2$$CH_2$; 3.03, s, *H*-$OCH_2CH_2$; ^{31}P{H} NMR (400 MHz, $CDCl_3$) δ (ppm): 20.69, 20.20, 11.03, 10.85, -5.45; ^{13}C NMR (400 MHz, $CDCl_3$) δ (ppm): 164.84 and 164.25 (carbonyl group), 132.52-125.01 aromatic carbon atoms of

BHET and PPD, 66.95 (HOCH$_2$*C*H$_2$-), 66.05 (-*C*H$_2$CH$_2$O-P(O)-), 62.98, d, ^{3}J(P,C)=6 Hz (-CH$_2$*C*H$_2$O-P(O)-); 59.86 (HO*C*H$_2$CH$_2$-);

S1.2. Interaction of BHET with PPD in the presence of HCl acceptor at a molar ratio 2:1 (model reaction 2). Product 2BPClTEA.

The reaction was carried out under the same experimental conditions as model reaction 1 with the following exceptions: molar ratio BHET/PPD=2:1, therefore the amount of BHET was 0.200 g, (0.0008 mol); the amount of TEA was 0.081 g, (0.0008 mol); THF – 20 ml. The isolated white, soft product was labeled **2BPClTEA** and characterized by ^{1}H and ^{31}P{H} NMR analyses. ^{1}H NMR (400 MHz, CDCl$_3$) δ (ppm): 8.04-7.31, m, Ar-*H*; 4.45, t, HOCH$_2$C*H*$_2$-OC(O), 4.41-4.22, m, –C*H*$_2$C*H*$_2$O-P(O)-OC*H*$_2$C*H*$_2$; 3.90, t, ^{3}J(H,H)=4 Hz, -HOC*H*$_2$CH$_2$; 3.05, s, *H*-OCH$_2$CH$_2$; ^{31}P{H} NMR (400 MHz, CDCl$_3$) δ (ppm): 20.20;

S1.3. Interaction of BHET with TMP at a molar ratio 1:2 (model reaction 3). Products BTMP5 and BTMP9.

1.000 g BHET (0.0039 mol) and 10.926 g, (0.0078 mol) of TMP were mixed in a three- necked round bottom flask equipped with an argon purging capillary, a magnetic stirrer, a condenser and thermometer. The reaction was carried out at 190°C with continuous stirring for 5 h. Termination of the release of methanol was the indicator that the reaction was completed. The content of the flask was allowed to cool down to room temperature. The product was dried under reduced pressure to get a semi-transparent yellowish, soft product; labeled **BTMP5** and characterized by ^{1}H, ^{31}P{H} and ^{31}P NMR analyses.

^{1}H NMR (400 MHz, CDCl$_3$) δ (ppm): 8.03-8.01, m, Ar-*H*; 4.42-4.29, m, -C(O)OC*H*$_2$- and P(O)O-C*H*$_2$-CH$_2$-; 3.86, s, C*H*$_3$OC(O)-Ar-; 3.69, d, ^{3}J(P,H)=12 Hz, P(O)O-C*H*$_3$; 3.66, d, ^{3}J(P,H)=12 Hz, P(O)O-C*H*$_3$; 3.34, s, -CH$_2$CH$_2$-O*H* ; ^{31}P{H} NMR (400 MHz, CDCl$_3$) δ (ppm): 2.63, 2.30; ^{31}P

NMR (400 MHz, $CDCl_3$) δ (ppm): 2.63, m, $^3J(P,H)$=11.4 Hz, $(OCH_3)_2P(O)CH_2CH_2$-O(O)C-Ar-C(O)O-CH_2CH_2OH; 2.30, m, $^3J(P,H)$=11.4 Hz, $(OCH_3)_2P(O)CH_2CH_2$-O(O)C-Ar-C(O)O-CH_2CH_2-O(O)P$(OCH_3)_2$;

The product BTMP5 was further heated at 190°C for additional 4 h. The obtained product was cooled down to room temperature, dried under dynamic vacuum to constant weight, labeled **BTMP9** and characterized by ^1H, $^{31}P\{H\}$, ^{31}P ,^{13}C NMR and TGA analyses.

^1H NMR (400 MHz, $CDCl_3$) δ (ppm): 8.03-8.01, m, Ar-*H*; 4.42-4.28, m, -C(O)OCH_2- and P(O)O-CH_2-CH_2-; 3.86, s, CH_3OC(O)-Ar-; 3.70, d, $^3J(P,H)$=12 Hz, P(O)O-CH_3; 3.66, d, $^3J(P,H)$=12 Hz, P(O)O-CH_3; 3.34, s, -CH_2CH_2-O*H*; $^{31}P\{H\}$ NMR (400 MHz, $CDCl_3$) δ (ppm): 2.70, 2.29; ^{31}P NMR (400 MHz, $CDCl_3$) δ (ppm): 2.70, m, $^3J(P,H)$=11.74 Hz, $(OCH_3)_2P(O)CH_2CH_2$-O(O)C-Ar-C(O)O-CH_2CH_2OH; 2.29, m, $^3J(P,H)$=11.74 Hz, (OCH3)$_2$P(O)CH_2CH_2-O(O)C-Ar-C(O)O-CH_2CH_2-O(O)P(OCH3)$_2$; ^{13}C NMR (400 MHz, $CDCl_3$) δ (ppm): 166.16 and 165.43, *C*=O; 133.70 and 129.65, aromatic carbon atoms; 70.41, C(O)OCH_2-; 63.81, d, $^2J(P,C)$=5.7 Hz, P(O)O-CH_2CH_2; 59.04, HOCH_2 ;54.46, d, $^3J(P,C)$=6.0 Hz, P-OCH_3; 54.12,d, $^2J(P,C)$=6.0 Hz, P-OCH_3; 52.42, CH_3OC(O)-Ar-;

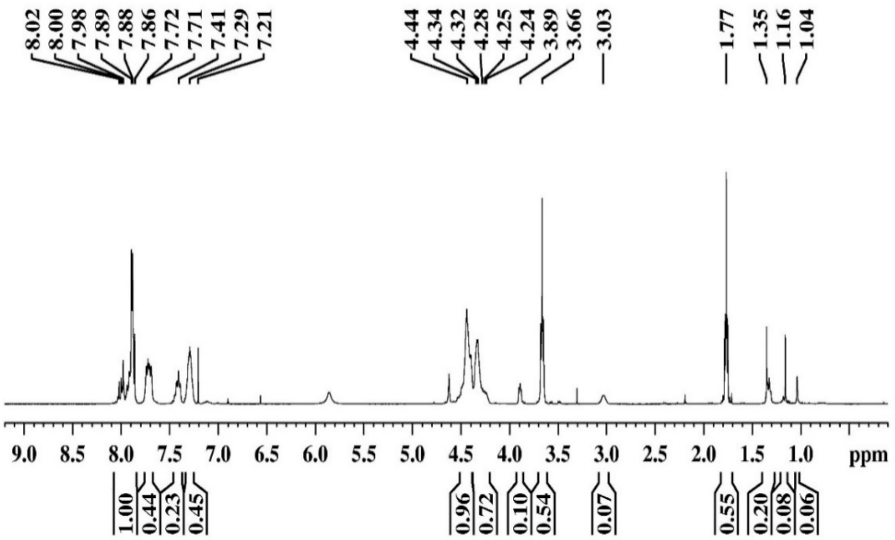

Figure S1. ^{1}H NMR spectrum of BPClTEA.

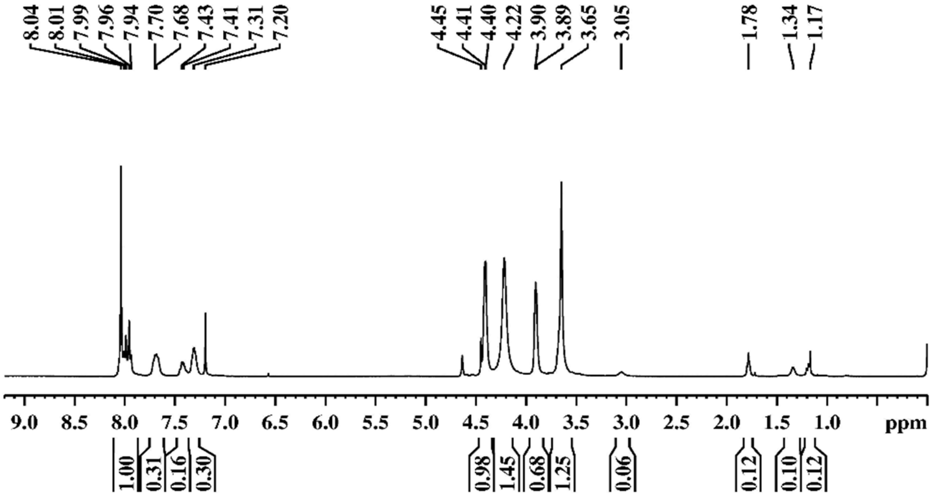

Figure S2. ^{1}H NMR spectrum of 2BPClTEA.

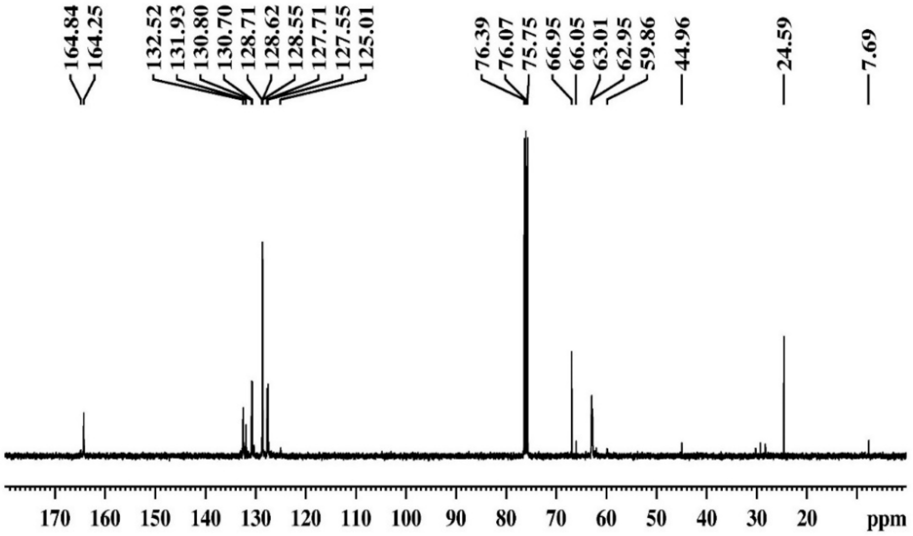

Figure S3. ^{13}C NMR spectrum of BPClTEA.

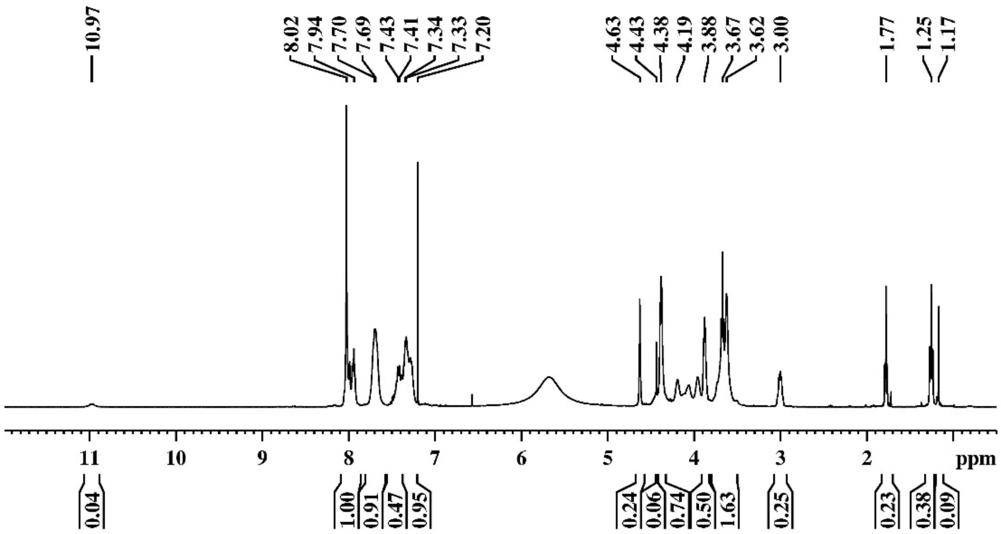

Figure S4. ^{1}H NMR spectrum of GP-PET/PPD.

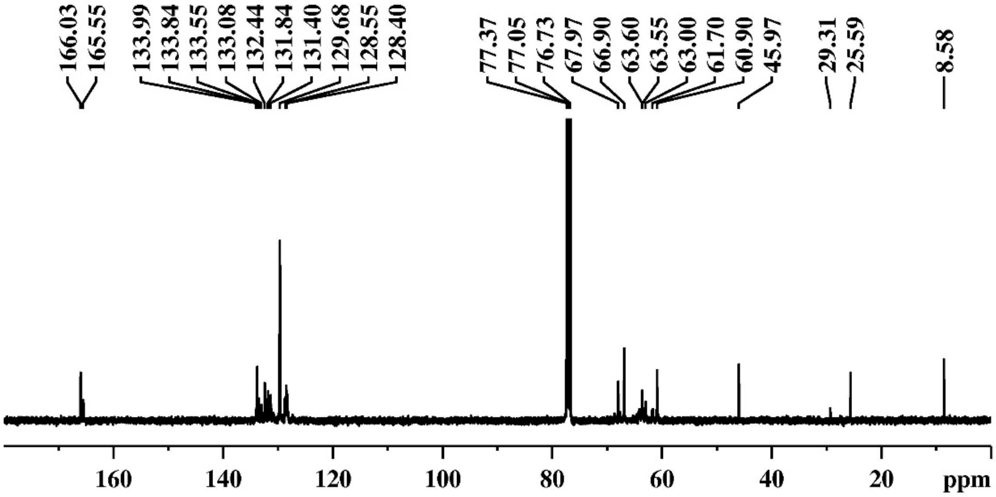

Figure S5. ^{13}C NMR spectrum of GP-PET/PPD.

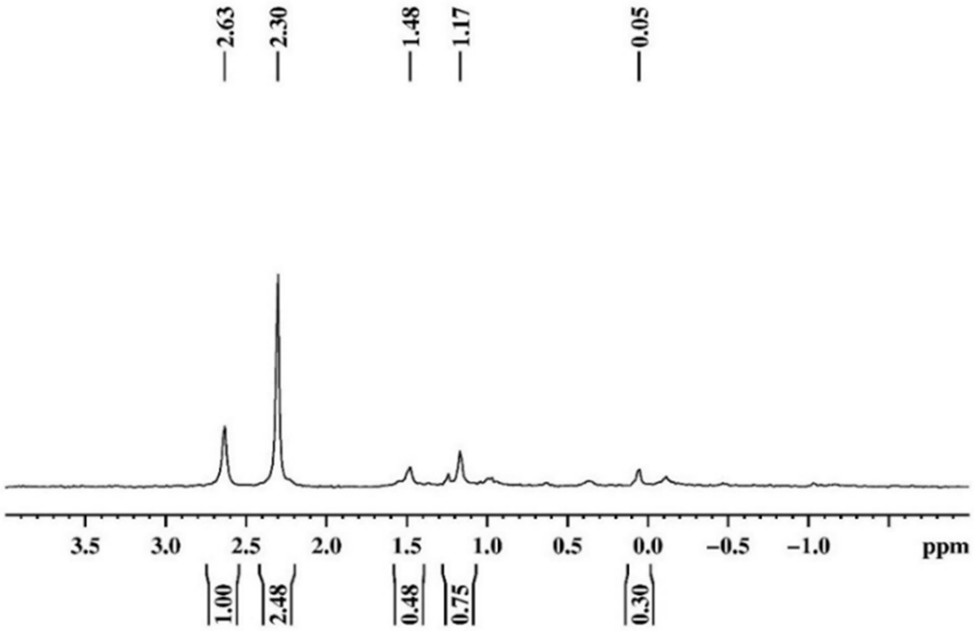

Figure S6. $^{31}P\{H\}$ NMR spectrum of BTMP5.

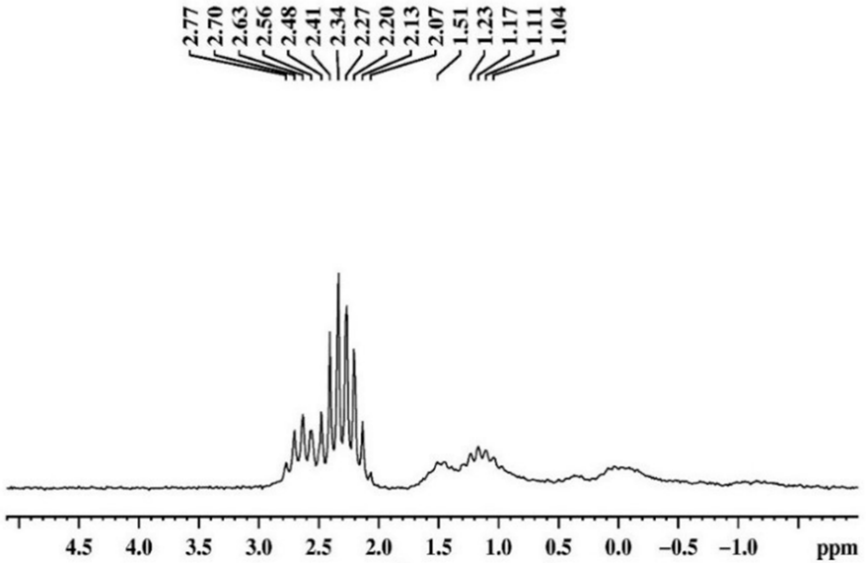

Figure S7. ^{31}P NMR spectrum of BTMP5.

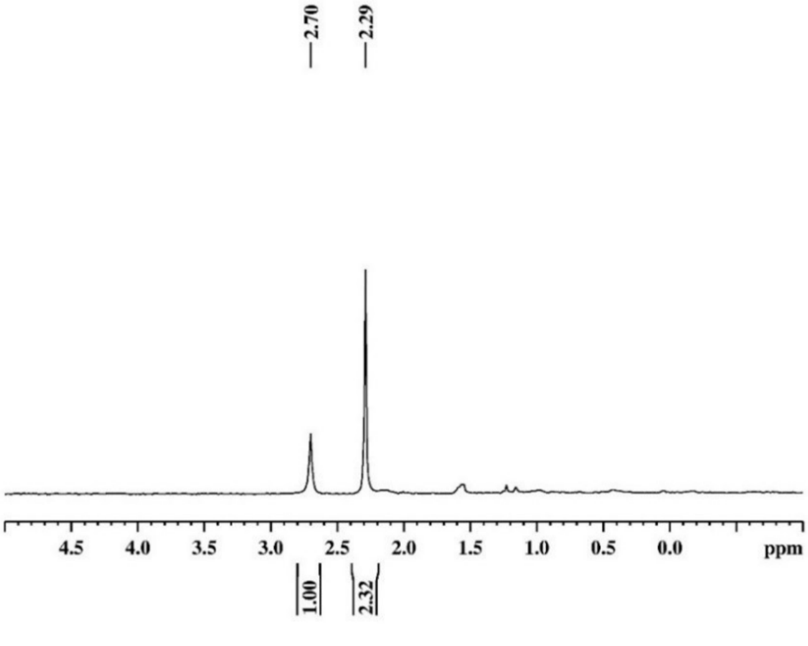

Figure S8. $^{31}P\{H\}$ NMR spectrum of BTMP9.

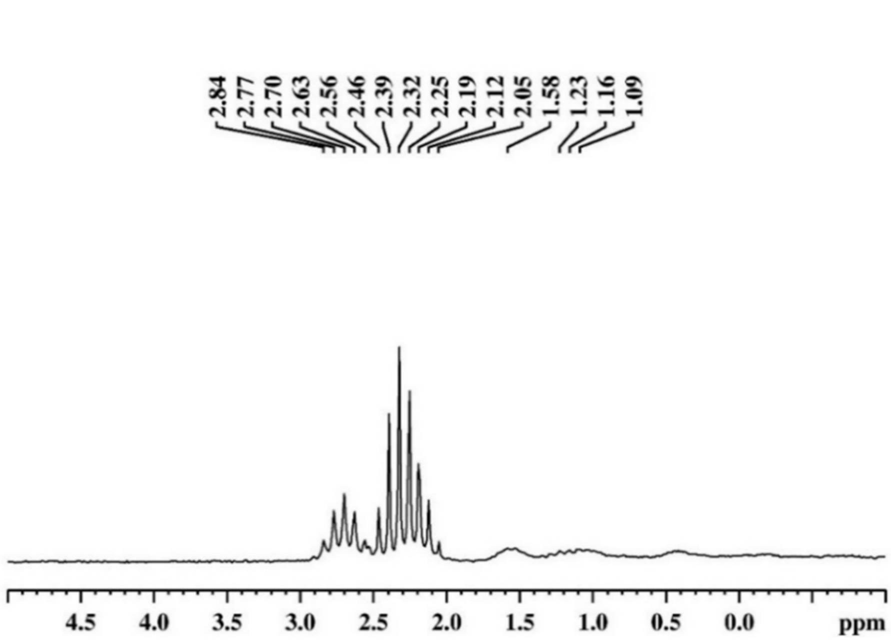

Figure S9. ^{31}P NMR spectrum of BTMP9.

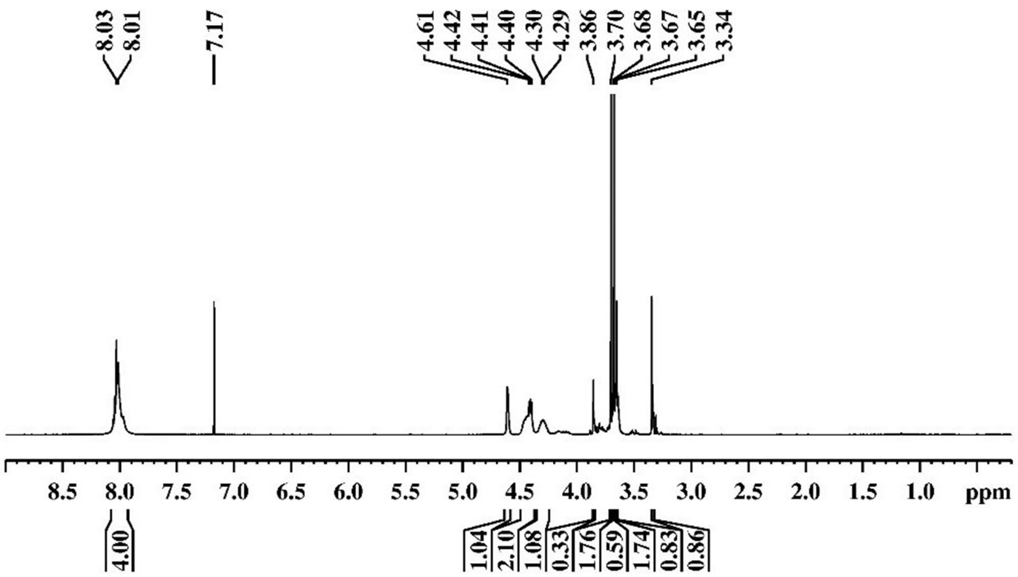

Figure S10. ^{1}H NMR spectrum of BTMP5.

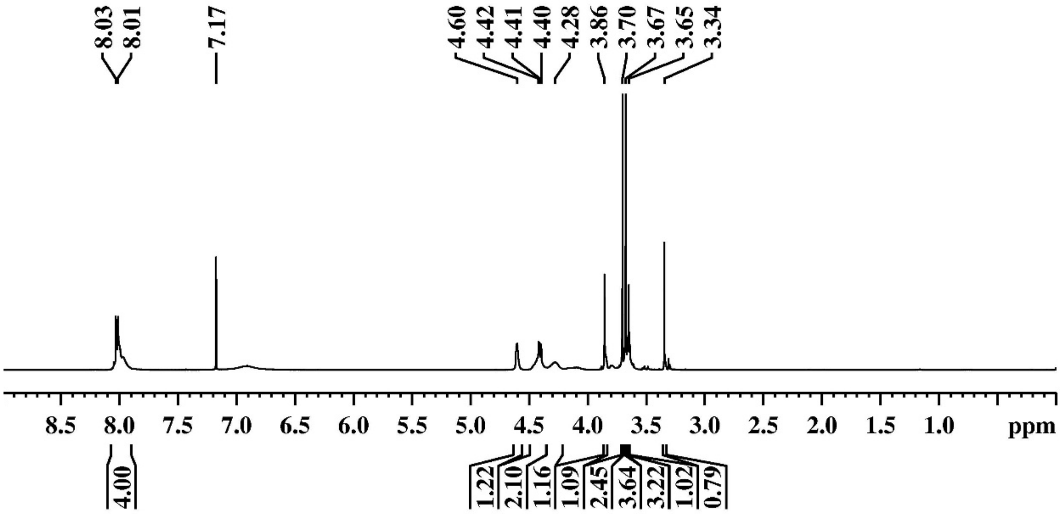

Figure S11. ^{1}H NMR spectrum of BTMP9.

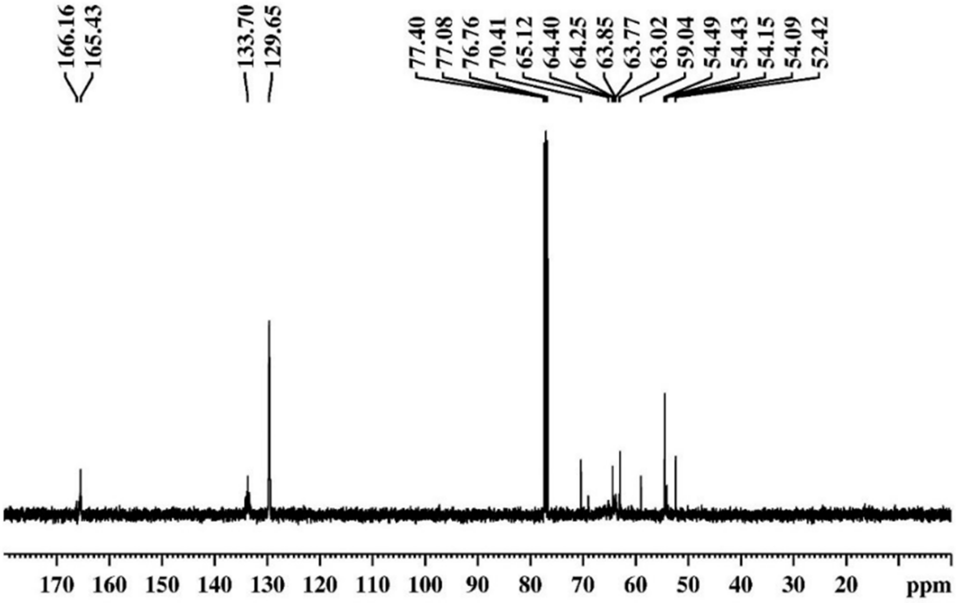

Figure S12. ^{13}C NMR spectrum of BTMP9.

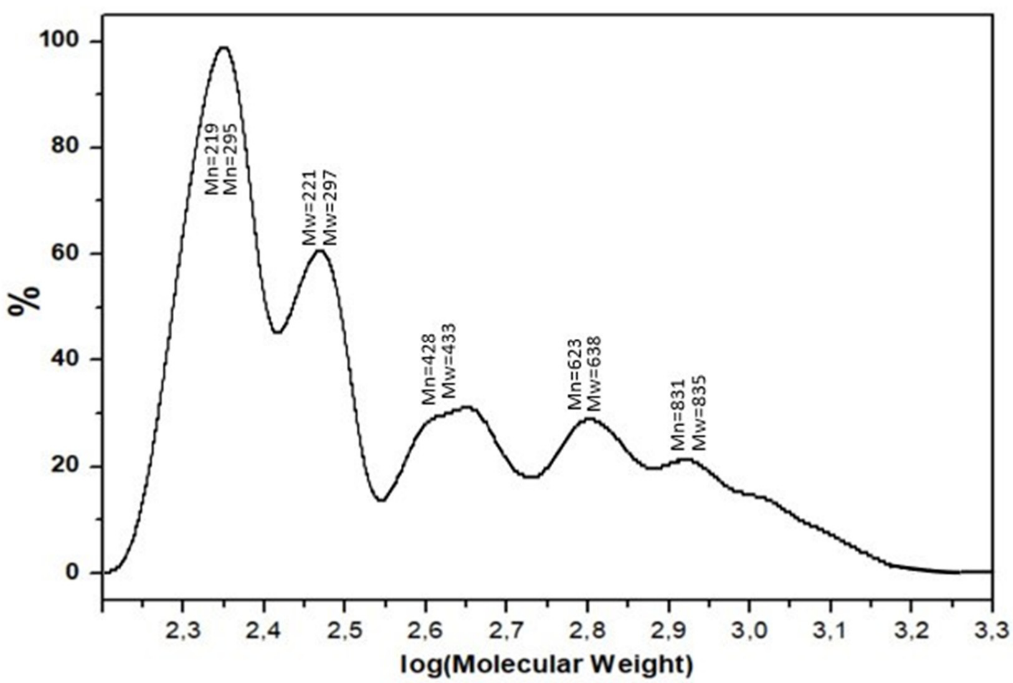

Figure S13. Molecular weight distribution according to GPC analysis of GP-PET/TMP.

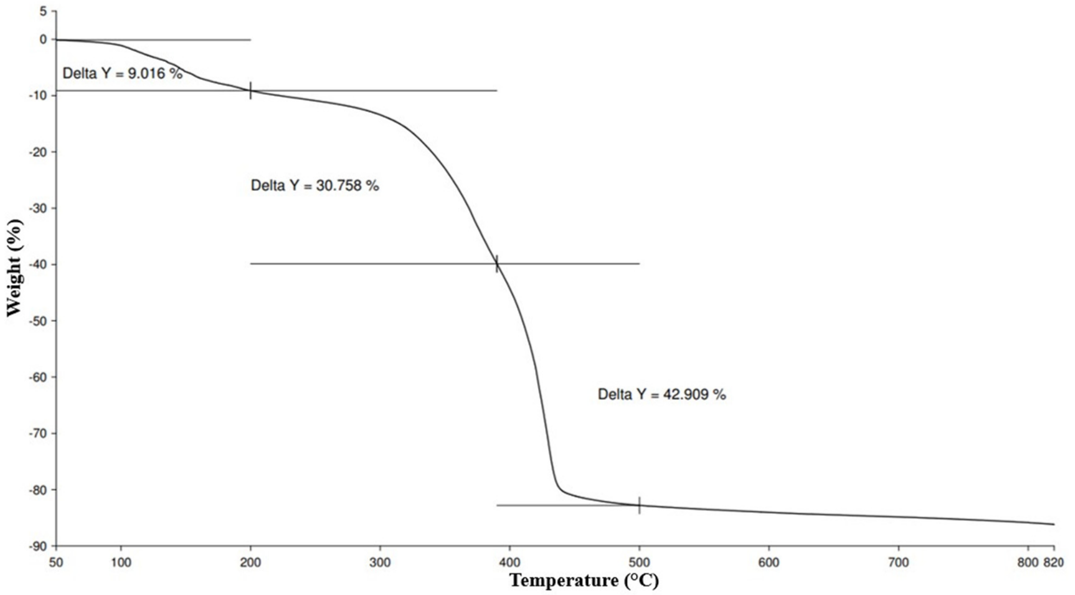

Figure S14. TGA curve of BPCTEA.

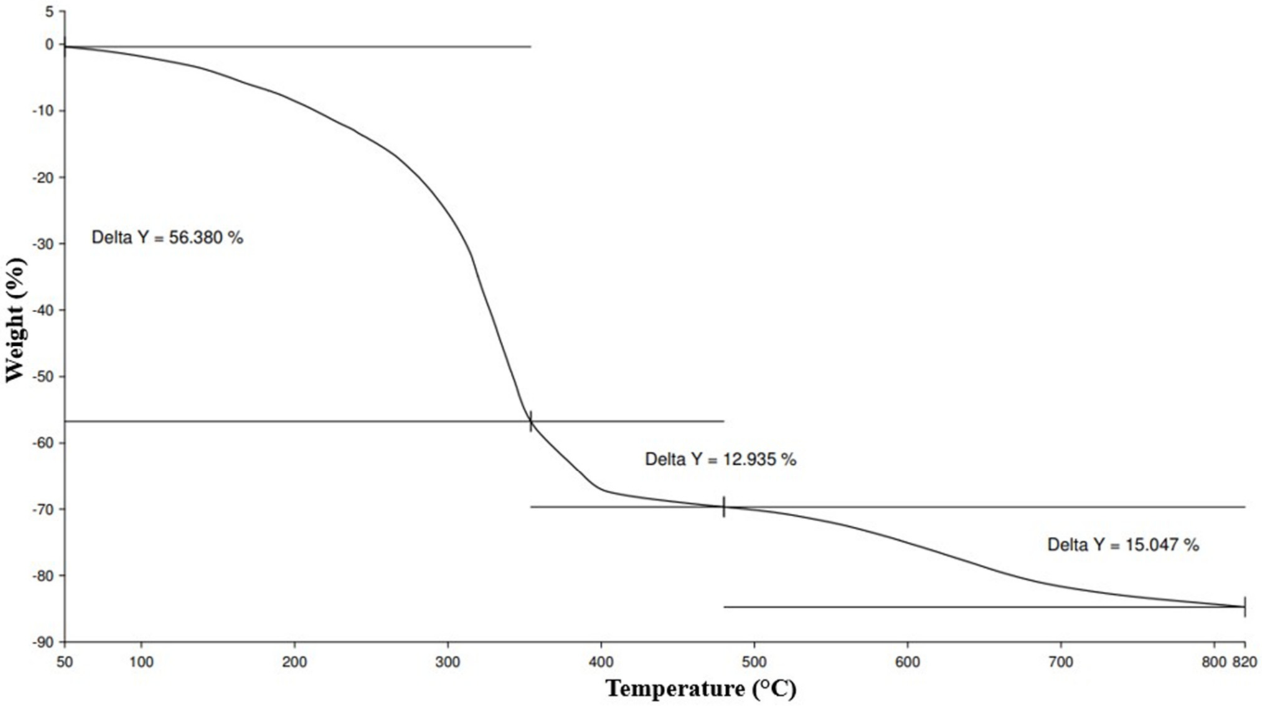

Figure S15. TGA curve of BTMP9.
